# Non-cirrhotic Ascites: A Case of Severe Alcoholic Hepatitis

**DOI:** 10.7759/cureus.58187

**Published:** 2024-04-13

**Authors:** Mohamed A Ebrahim, Eli A Zaher, Parth Patel, Marwan K Ahmed, Kanwal Ahmed

**Affiliations:** 1 Internal Medicine, Ascension Saint Joseph - Chicago, Chicago, USA

**Keywords:** alcoholic liver disease, mdf, meld-na, alcoholic hepatitis, ascites

## Abstract

This case report presents a unique instance of ascites in acute alcoholic hepatitis (AH) occurring in a non-cirrhotic patient. Comprehensive diagnostic evaluation excluded alternative etiologies, pinpointing sinusoidal non-cirrhotic portal hypertension. Present therapeutic modalities for AH, including steroids and pentoxifylline, offer limited efficacy, necessitating ongoing investigation. Liver transplantation may be contemplated in refractory cases. This case underscores the intricate nature of AH presentations and the challenges in their management, emphasizing the imperative need for continued research to delineate optimal therapeutic strategies. Early intervention remains pivotal in addressing AH complications, underscoring the need for heightened clinical vigilance and proactive treatment approaches in such cases.

## Introduction

Alcoholic liver disease (ALD) is the leading cause of alcohol-related death worldwide. It encompasses a spectrum of pathology, ranging from steatosis to cirrhosis and subsequent hepatocellular carcinoma. Alcoholic hepatitis (AH) is an acute hepatic inflammation that manifests in individuals with either steatosis or pre-existing cirrhosis [1]. It accounts for about four of every 100,000 hospital admissions in the United States. Severe cases of AH may present with jaundice, hepatic encephalopathy, coagulopathy, and ascites [2]. While underlying cirrhosis increases the likelihood of ascites development in AH, it is rarely seen in those without pre-existing advanced liver disease [3]. We present a rare finding of ascites in the setting of AH in a non-cirrhotic patient.

## Case presentation

A 36-year-old non-obese female with a history of alcohol use disorder presented to the emergency department with complaints of constipation, abdominal distension, productive cough, and non-bloody vomiting for the preceding week. She denied having similar symptoms in the past but endorsed an escalation in her drinking habits two weeks before the hospital visit. She likewise denied having fevers, chills, abdominal pain, dark stools, bloody stools, changes to her menstrual cycle, or any history of heart disease or clotting disorder. No use of home medications was reported. 

Physical examination on admission revealed a diffusely distended, hard, and tender abdomen with a positive fluid wave. The skin evaluation revealed extensive jaundice. Alertness and orientation were adequate although she was noticed to be confused with slow speech. Vital signs on admission were positive for tachycardia to 105 bpm but were otherwise normal. Initial blood work is presented in Table 1. The model for end-stage liver disease with sodium (MELD-Na) score was 24 and Maddrey’s discriminant dunction (MDF) was 42.2. 

**Table 1:** Admission laboratory workup

Component	Result	Reference range
Hemoglobin (g/dL)	12.5	12.0-15.3
White cell count (k/mm cu)	8.7	4.0-11.0
Mean corpuscular volume (f/L)	107	80.0-100.0
Platelets (k/mm cu)	238	150-450
Creatinine (mg/dL)	0.37	0.6-1.2
Sodium (mmol/L)	134	133-144
Albumin (g/dL)	3.0	3.5-5.7
Aspartate aminotransferase (IU/L)	153	13-39
Alanine aminotransferase (IU/L)	43	7-52
Total bilirubin (mg/dL)	9.1	0.0-1.0
Alkaline phosphatase (IU/L)	186	35-104
International normalized ratio	1.7	0.9-1.1
Ammonia (UMOL/L)	76	16-52
Lipase (IU/L)	14	11-82

**Figure 1:**
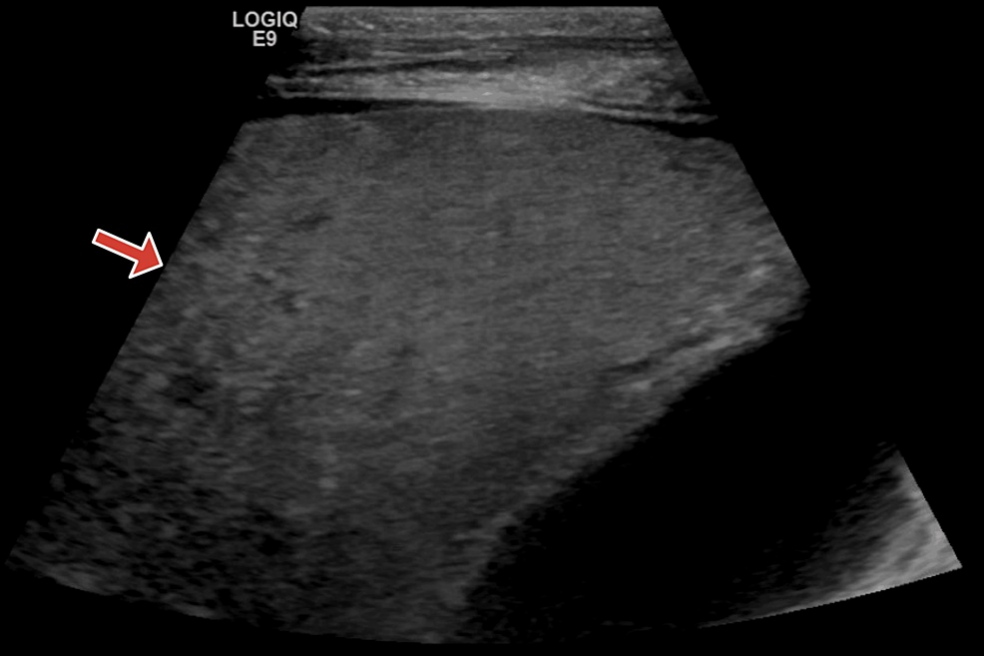
Liver ultrasound Red arrow: Mild hepatomegaly with diffuse hepatic steatosis without evidence of cirrhosis

On consulting gastroenterology, it was recommended that the patient be started on scheduled lactulose and ceftriaxone. Corticosteroids were deferred due to concern for aspiration pneumonia on chest X-ray. Further workup for viral hepatitis, autoimmune hepatitis, Wilson’s disease, and hemochromatosis was negative. Liver ultrasound with Doppler was consistent with diffuse steatosis and ascites without evidence of cirrhosis or thrombosis (Figure 1). Echocardiography was negative. Paracentesis withdrew two liters of fluid. Peritoneal fluid analysis was negative for peritonitis based on negative gram stain and culture. The ascites was consistent with a portal hypertensive etiology given its serum ascites albumin gradient (SAAG) of 2.5 g/dL.

The patient’s condition improved rapidly thereafter, and she was discharged home with instructions to quit alcohol, limit dietary sodium, and use daily lasix. She was further instructed to schedule a visit with gastroenterology and an outpatient MRI of her abdomen but was lost to follow-up.

## Discussion

Alcoholic liver disease encompasses a range of liver injuries, spanning from simple steatosis to AH to cirrhosis [1]. The pathogenesis of ALD is complex, involving genetic predisposition, alcohol-induced hepatocyte damage, reactive oxygen species, and gut-derived microbial components, leading to steatosis and inflammatory cell recruitment in the liver. Ongoing alcohol consumption and pro-inflammatory cytokines further activate stellate cells, driving progressive fibrosis. Despite undergoing treatment and abstaining from alcohol, many patients suffering from severe AH fail to achieve recovery [4]. If improvement isn't observed within three months despite these interventions, the likelihood of spontaneous recovery diminishes, increasing the risk of mortality. Persistent alcohol consumption triggers fatty liver changes, which can escalate into inflammation, fibrosis, and ultimately cirrhosis if excessive drinking continues [5]. Alcoholic hepatitis manifests as acute hepatic inflammation, posing significant morbidity and mortality risks, particularly in individuals with pre-existing steatosis or cirrhosis. Furthermore, AH has the potential to advance to alcohol-induced liver cirrhosis, lead through portal hypertension, involving major complications of hepatic encephalopathy, life-threatening variceal bleeding, and recurrent ascites [1,2].

Ascites occurs when non-hemorrhagic fluid accumulates in the abdominal cavity. While liver cirrhosis is the main cause, various other conditions have been linked to non-cirrhotic ascites. In 20% of cases, ascites is unrelated to liver cirrhosis, with distinct pathophysiological mechanisms at play [3]. Non-cirrhotic ascites usually stems from non-cirrhotic portal hypertension (NCPH), which encompasses a diverse range of liver conditions primarily impacting the vascular system [6]. These disorders are anatomically categorized based on the site where blood flow resistance occurs, including pre-hepatic, hepatic, and post-hepatic classifications. Hepatic causes are further subclassified histologically as pre-sinusoidal, sinusoidal, and post-sinusoidal causes. Pre-sinusoidal factors include porto-sinusoidal vascular disease (PSVD), portal vein obstruction (neoplastic and non-neoplastic), and schistosomiasis. Sinusoidal causes encompass drug-induced effects, severe alcoholic liver damage, viral hepatitis, and amyloidosis, among others. Post-sinusoidal factors involve conditions such as Budd-Chiari syndrome, veno-occlusive disease, and primary vascular malignancies, along with several others including hypervitaminosis A and Gaucher’s disease [7]. These diverse etiologies contribute to the complex landscape of NCPH. Per this case report, acute alcoholic intoxication, especially in individuals with pre-existing steatosis, triggers rapid liver function decompensation, resulting in sinusoidal NCPH. This condition subsequently led to the development of ascites in our patient.

Concerning available treatment for AH, recent trials, such as the steroids or pentoxifylline for alcoholic hepatitis (STOPAH) trial and a meta-analysis examining the efficacy of steroids and pentoxifylline, have shown only short-term mortality benefits (at 28 days) with steroid treatment, and without any significant differences in mortality rates at six months or one year. Pentoxifylline usage did not demonstrate any benefits. The exclusion of patients with renal dysfunction in the trial might have biased results against pentoxifylline, as its benefits were previously associated with preventing or reversing hepatorenal syndrome. Treatment attempts with anti-tumor necrosis factor (TNF) agents like infliximab and etanercept haven't shown survival benefits and could potentially worsen outcomes [8]. Patients with AH face an increased risk of infections, especially when undergoing steroid treatment, which could exacerbate complications such as acute renal injury and multi-organ dysfunction, ultimately affecting prognosis. Monitoring daily caloric intake is crucial, with nutritional supplementation recommended if oral intake falls below 1200 kcal per day, preferably administered orally or through a nasogastric tube [9]. Liver transplantation may be considered for non-responsive cases to steroids with a MELD score exceeding 26. However, several barriers, including concerns about relapse, organ scarcity, and social and ethical considerations, exist [10]. A 2015 survey of liver transplant programs revealed that only 27% of programs offered transplants to patients with AH, with a mere 1.37% of liver transplants performed on such patients. Nevertheless, survival rates at six months, one year, and five years were comparable to those with similar MELD scores, with recidivism rates also similar to those transplanted for alcohol-related cirrhosis [11]. 

## Conclusions

Alcoholic hepatitis leading to ascites in a non-cirrhotic setting presents a complex and clinically significant scenario. The presence of ascites in non-cirrhotic AH signifies severely damaged liver causing further inflammation and subsequent dysfunction, indicating a poor prognosis and heightened risk of disease progression. Management requires a multidisciplinary approach focusing on addressing liver inflammation, managing complications such as ascites, and implementing interventions for alcohol cessation and supportive care. Early recognition and comprehensive management are crucial for optimizing outcomes in these patients and preventing disease progression.
